# Differential Spleen miRNA Expression Profile of Beagle Dogs Infected with *Toxocara canis*

**DOI:** 10.3390/ani12192638

**Published:** 2022-09-30

**Authors:** Yue Xu, Hao-Yu Li, Lang Cai, Shi-Chen Xie, Yang Zou, Xing-Quan Zhu, Wen-Bin Zheng

**Affiliations:** 1 Laboratory of Parasitic Diseases, College of Veterinary Medicine, Shanxi Agricultural University, Jinzhong 030801, China; 2 State Key Laboratory of Veterinary Etiological Biology, Lanzhou Veterinary Research Institute, Chinese Academy of Agricultural Sciences, Lanzhou 730046, China

**Keywords:** transcriptomics, miRNA, *Toxocara canis*, spleen, Beagle dog

## Abstract

**Simple Summary:**

As one of the neglected parasitic zoonoses, toxocariasis has gained increasing international attention. To date, however, little is known about the spleen microRNAs (miRNA) expression profiling in definitive or paratenic hosts after *Toxocara canis* infection. In this study, we conducted sequencing-based transcriptome profiling of miRNA expression in the spleen of Beagle puppies after *T. canis* infection to understand the potential biological roles of the altered miRNAs. Some differentially expressed miRNAs (DEmiRNAs) were identified after *T. canis* infection. The transcriptional profiling data suggested that the DEmiRNAs play significant roles in the pathogenesis of toxocariasis.

**Abstract:**

*Toxocara canis* is an unnoticed zoonotic helminth that causes severe disease in animals and humans. The spleen has a wide range of immunological functions in protecting the host against infection by many pathogens, but the function of the spleen in *T. canis* infection is still to be clarified, especially for the role of spleen microRNAs (miRNAs). In this study, deep sequencing of spleen RNA samples of 18 Beagle puppies was conducted to uncover the miRNAs expression profiling at 24 h post-infection (hpi), 96 hpi, and 36 days post infection (dpi). A total of 20, 34, and 19 differentially expressed miRNAs (DEmiRNAs) were identified at 24 hpi, 96 hpi, and 36 dpi, respectively. These DEmiRNAs (e.g., cfa-miR-206, cfa-miR-331, and cfa-miR-339) could play critical roles in Beagle puppies against *T. canis* infection, such as influencing inflammatory and immune-related cells and cytokines, by regulating target genes that are tightly associated with host immune function and enriched in immune response and immune pathways based on GO annotation and KEGG enrichment analysis. The current study discovered marked alterations of spleen miRNAs after *T. canis* infection, with potential effects on the pathogenesis of toxocariasis.

## 1. Introduction

*Toxocara canis*, the key member of the genus *Toxocara,* causes severe disease in humans and animals, which has led to increasing international attention [1,2]. According to the US Centers for Disease Control and Prevention (CDC), toxocariasis was ranked as one of the six most neglected parasitic infections in public health (https://www.cdc.gov/parasites/npi/, accessed on 26 August 2022). *T. canis* does not develop into adults in intermediate hosts, such as humans, but causes various larval migrations, while, in the small intestine of the definitive host, *T. canis* develops into an adult, and the eggs are excreted into the environment in the feces [3]. However, the mechanisms of host-*T. canis* interactions are not fully clear, such as how *T. canis* evades the host’s immune surveillance. The understanding of pathogen–host interactions and disease ecology is changing with the advent of new molecular tools, such as high-throughput transcriptomics sequencing [4]. Large sets of novel and known microRNAs (miRNAs) and their target genes involved in innate and adaptive immune responses have been discovered following the rapid advancement of next-generation sequencing (NGS) techniques [5]. At the post-transcriptional level, miRNAs (~22 bp) that regulate gene expression by binding to the 3’-untranslated region (UTR) of the target gene are an important class of small non-coding RNAs [6], which can also silence protein expression to mediate mRNA expression via cleavage and degradation of the mRNA transcript or by inhibiting translation [7]. It is becoming increasingly clear that parasites can produce miRNAs to regulate the expression of host miRNAs for their own survival [8].

A previous study revealed significant changes in miRNA expression profiles in Beagle puppies’ lives, as well as the roles of some miRNAs in a dynamic equilibrium between anti- and pro-inflammatory responses [9]. The spleen is the site of settlement of T cells and B cells and has a wide scope of immunological functions [10]. Therefore, in our previous study, we used RNA-seq to identify significant alterations in the expression patterns of messenger RNAs (mRNAs) and long non-coding RNAs (lncRNAs) in Beagle puppies’ spleen to reveal the role of differentially expressed mRNAs and lncRNAs in the spleen after *T. canis* infection [11]. Nevertheless, little is known about the changes of miRNA expression in dog splenic response to *T. canis* infection. Thus, to better understand the altered miRNAs, we undertook a sequencing-based transcriptomic analysis of miRNA expression in Beagle puppies’ spleen after *T. canis* infection at 24 h post-infection (hpi), 96 hpi, and 36 days post infection (dpi).

## 2. Materials and Methods

### 2.1. Ethics Approval

All animal experiments were approved by the Animal Ethics Committee of Lanzhou Veterinary Research Institute, Chinese Academy of Agricultural Sciences (Approval no: 2018-015), and all experiments were performed in accordance with the Animal Ethics Procedures and Guidelines of the People′s Republic of China.

### 2.2. Animal Infection and Sample Collection

Eighteen 6–7-week-old *Toxocara*-naive, gender-neutral, specific pathogen-free (SPF) Beagle puppies were used and raised in the National Canine Laboratory Animal Resource Center without vaccination and feeding any medication in this study. The rearing environment has been described in a previous study [12]. In brief, these puppies were randomly divided into three groups according to the time point at which the larvae migrate and develop in the definitive host: 24 hpi (liver infection stage), 96 hpi (lung infection stage), and 36 dpi (small intestine infection stage). Each group of Beagle puppies came from the same litter to reduce background differences, including three infected puppies and three control puppies. Procedures regarding infection, anesthesia, euthanasia, and spleen acquisition and storage were described in a previous study [11]. Briefly, Beagle puppies in the infected and control groups were orally infected with 300 embryonated *T. canis* eggs and equal amounts of saline, respectively. Puppies were euthanized by injecting potassium chloride (KCl) solution under general anesthesia with Zoletil™ 50 (Virbac, Carros, France). The collected splenic tissues were frozen in liquid nitrogen. None of the puppies, including infected puppies and control puppies, had any negative health effects before being euthanized.

### 2.3. RNA Extraction and RNA Sequencing

The total RNA of the 18 puppy spleen tissues was extracted using TRIZOL (Life Technologies, Carlsbad, CA, USA). In brief, 100 mg of frozen tissue was powdered in liquid nitrogen, and then 1 mL TRIzol was added to solubilize the tissue for five minutes at room temperature. Subsequently, 0.2 mL of chloroform was added and mixed vigorously by vortex for 15 s; then, the mixture was stood for 2–3 min and was centrifuged at 12,000× *g* for 10 min at room temperature. Subsequently, the upper clear phase per sample was carefully absorbed and transferred to a new tube, followed by adding 0.5 mL isopropanol per each milliliter and was mixed vigorously by vortex, then the mixture was stood for 10 min and was centrifuged at 12,000× *g* for 10 min at 4 °C to collect the precipitated RNA. Then, the supernatant was carefully decanted, and the pellet was immediately resuspended in 1X SDS solubilization buffer with NaOAc to three M. The RNA was re-extracted with phenol and precipitated with 75% ethanol at 7500× *g* for five min at 4 °C to collect total RNA. The genomic DNA was removed by using DNase I (NEB, Ipswich, MA, USA). In brief, the total RNA was incubated in one μL 10X DNase I Buffer, one μL DNase I, and eight μL RNase-Free Water for 15 min at room temperature to remove genomic DNA. Then, 1 μL of 25 mM EDTA solution was added to the reaction and heated at 65 °C for 10 min to heat inactivation of the DNase I. RNA degradation and contamination were examined on 1% agarose gels for preliminary detection, and sample with distinct 18S and 28S bands and without significant stray bands was considered as high-quality RNA. RNA purity was examined using the NanoPhotometer^®^ spectrophotometer (IMPLEN, Westlake Village, CA, USA), and sample with an OD260/280 value of around 2.0 was considered high-quality RNA. Utilizing the Qubit^®^ RNA Assay Kit in Qubit^®^ 2.0 Fluorometer (Life Technologies, CA, USA), RNA concentration was measured. RNA integrity was evaluated using the RNA Nano 6000 Assay Kit of the Agilent Bioanalyzer 2100 system (Agilent Technologies, Santa Clara, CA, USA). High-quality total RNA samples with RNA integrity number (RIN) >8.0 were used to construct subsequent small RNA sequencing libraries.

Then, 3 μg total RNA was used as the input material, and NEBNext^®^Multiplex Small RNA Library Prep Set for Illumina^®^ (NEB, USA) was used to generate the small RNA sequencing libraries, followed by adding index codes to attribute sequences to each sample. First-strand cDNA was synthesized by using M-MuLV Reverse Transcriptase (RNase H-), and then PCR amplification was carried out by utilizing the LongAmp Taq 2X Master Mix, SR Primer for Illumina, and index (X) primer. PCR products were purified using an 8% polyacrylamide gel (100 V, 80 min). DNA fragments of 140–160 bp were dissolved in eight μL elution buffer. Finally, the quality of the libraries was evaluated on the Agilent Bioanalyzer 2100 system. The index-coded samples were clustered on a cBot Cluster Generation System using the TruSeq SR Cluster Kit v3-cBot-HS (Illumina, San Diego, CA, USA). Afterward, the small RNA libraries were sequenced on Illumina Hiseq 2000 platform, generating 50 bp single-end reads.

### 2.4. Identification of miRNAs and Differentially Expressed miRNAs

Clean data (clean reads) were generated by removing some of the low-quality reads from the raw data, such as containing ploy-N and 5′adapter contaminants, without 3′adapter or the insert tag. Moreover, GC-content, error rate, and Q30 of the raw data were calculated. To analyze the expression and distribution of the known miRNAs, the reference data of *Canis lupus familiaris* were downloaded from the Ensembl database (CanFam3.1) and mapped by Bowtie [13]. Custom scripts were used to obtain the number of known miRNAs. sRNA-tools-cli and modified software mirdeep2 [14] were used to obtain the potential miRNAs and draw the secondary structures based on the MiRBase20.0. Novel miRNAs were predicted by the hairpin structure of miRNA precursors, as well as exploring the secondary structure, the minimum free energy of small RNA tags unannotated in the previous steps, and the Dicer cleavage sites by integrating software mirdeep2 [14] and miREvo [15]. The expression levels of miRNAs were assessed by TPM (transcript per million) [16]. The DEGseq R package (3.0.3) was used to implement differential expression analysis between infected puppies and control puppies, and miRNAs with *p*-value < 0.05 were considered differentially expressed.

### 2.5. GO and KEGG Enrichment Analysis

GOseq software and KOBAS software were used to implement the GO annotation and KEGG enrichment analysis for the underlying target genes of differentially expressed miRNAs (DEmiRNAs) [17,18]. The screening criterion for significantly enriched GO terms and KEGG pathways was *p*-value < 0.05.

### 2.6. Real-Time Quantitative PCR

To validate the results of RNA-Seq, 15 DEmiRNAs (5 per infection stage) were randomly selected and their expression was analyzed by qRT-PCR on LightCycler 480 (Roche, Basel, Switzerland). The kit, amplification procedure, and melting curve analysis for qRT-PCR were conducted strictly according to the previous description [12]. Briefly, the validation was performed using a qRT-PCR kit (TianGen, Beijing, China). The amplification procedure was initial denaturation (95 °C, 15 min) and 45 cycles (94 °C, 20 s and 60 °C, 34 s). The melting curve analysis was 95 °C (10 s), 65 °C (1 min), and then gradually increased from 65 °C to 95 °C. U6, a class of nuclear small molecule RNA that is initiated by RNA polymerase III, was used as an internal control. The 15 DEmiRNAs and their corresponding amplification primers are shown in Table S1.

## 3. Results

### 3.1. Characteristic Features of RNA-Sequencing Data

**Figure 1:**
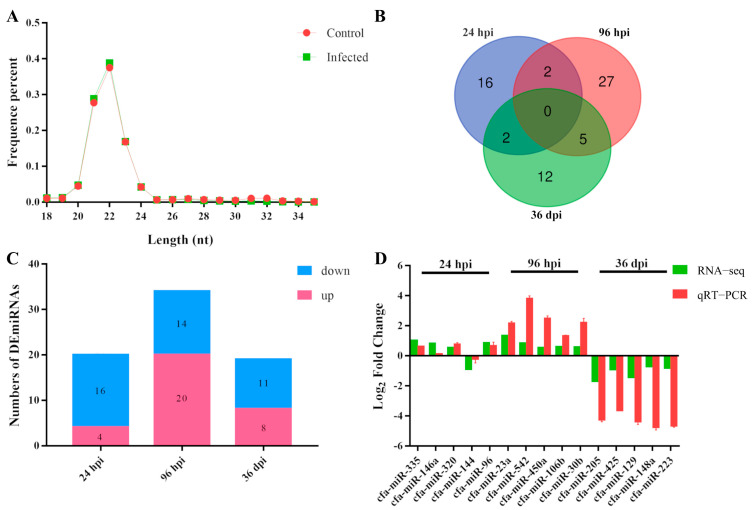
The length range and the differential expression of miRNAs in the spleen of puppies after *Toxocara canis* infection at 24 h post-infection (hpi), 96 hpi, and 36 days post-infection (dpi). (**A**) The length distribution range of identified miRNAs from the clean reads. (**B**) Venn diagrams showing the numbers of unique and common differentially expressed miRNAs (DEmiRNAs) in the spleen of Beagle puppies infected by *T. canis* at 24 hpi, 96 hpi, and 36 dpi. (**C**) DEmiRNA count for each of the three infection stages. The hues blue and pink represent the downregulated DEmiRNAs and upregulated DEmiRNAs, respectively. (**D**) qRT-PCR verification of DEmiRNA expression at 24 hpi, 96 hpi, and 36 dpi. The Y-axis represents the log_2_ (Fold Change) of DEmiRNAs, while the X-axis displays the investigated DEmiRNAs. The standard deviation (three replicates) is shown by the error bars.

In total, 12.722 G data were generated from 18 spleen samples of Beagle puppies, obtaining 254,435,186 raw reads. Among them, an average of 0.678 G data was generated in the control group, and 0.736 G data were generated in the infected group. The GC-content, error rate, and Q30 of raw reads were 49.28%, 0.01%, and 97.63%, respectively. After screening, a total of 252,067,690 clean reads, with 96.07% mapping into the reference genome, were obtained from 18 samples. Compared to the reference sequences, 233,314,009 clean reads were identified as sRNAs, among which 163,137,451 clean reads were identified as known miRNAs that accounted for 69.99% of the total sRNAs, and 1,863,930 clean reads were identified as novel miRNAs that accounted for 0.81% of the total sRNAs. Most of the small RNA fragments are concentrated between 20 nt and 24 nt, and 182 novel miRNAs and 314 known miRNAs were identified (Figure 1A).

### 3.2. Patterns of DEmiRNAs

Overall, 64 DEmiRNAs were identified in this study (Figure 1B). At 24 hpi, 20 DEmiRNAs were identified with 16 downregulated DEmiRNAs, and 4 upregulated DEmiRNAs; at 96 hpi, 34 DEmiRNAs were identified with 14 downregulated DEmiRNAs and 20 upregulated DEmiRNAs; at 36 dpi, 19 DEmiRNAs were identified with 11 downregulated DEmiRNAs and eight upregulated DEmiRNAs (Figure 1C; Table S2).

### 3.3. Quantitative RT-PCR Validation of RNA-Seq Results

The validity of the RNA-seq outcomes was verified independently using the qRT-PCR method. The qRT-PCR validation results of the 15 randomly selected miRNAs showed the same trend as the sequencing results, demonstrating the reliability of the sequencing results (Figure 1D).

### 3.4. Target Gene Prediction and Functional Analysis of DEmiRNAs

Overall, 8242 potential target mRNAs of 470 miRNAs were predicted, including 676 potential target mRNAs of 18 DEmiRNAs at 24 hpi; 1738 potential target mRNAs of 33 DEmiRNAs at 96 hpi; and 545 potential target mRNAs of 19 DEmiRNAs at 36 dpi (Table S3). The potential target genes of DEmiRNAs were enriched by GO annotation analysis (Table S4). The results showed that at 24 hpi, 497 potential target mRNAs (e.g., *irf1*, *tlr7,* and *masp1*) of 18 DEmiRNAs were enriched in 490 GO terms; of those, 316 GO terms belonged to biological process (e.g., receptor-mediated virion attachment to host cell, membrane organization, and carboxylic acid catabolic process), 64 GO terms belong to cellular component (e.g., vesicle, coated vesicle and cytoplasmic vesicle), and 110 GO terms belong to molecular function (e.g., clathrin adaptor activity, endocytic adaptor activity, and quinone cofactor methyltransferase activity). At 96 hpi, 1499 potential target mRNAs of 33 DEmiRNAs (e.g., *nlrp3*, *tnf,* and *tap1*) were enriched in 532 GO terms; of those, 368 GO terms belong to biological process (e.g., positive regulation of JUN kinase activity, forebrain neuron differentiation, and clathrin-mediated endocytosis), 47 GO terms belong to cellular component (e.g., dendritic spine head, postsynaptic endocytic zone, and postsynaptic endocytic zone membrane) and 117 GO terms belong to molecular function (e.g., ER retention sequence binding, carboxylic acid binding, and ErbB-3 class receptor binding). At 36 dpi, 521 potential target mRNAs (e.g., *il17f*, *sema7a*, and *il12b*) of 19 DEmiRNAs of were enriched in 552 GO terms; of those, 414 GO terms belong to biological processes (e.g., cardiolipin acyl-chain remodeling, regulation of mRNA processing and B-1 B cell homeostasis), 61 GO terms belong to cellular components (e.g., telomerase holoenzyme complex, cytoskeletal part, and cyclin E1-CDK2 complex), and 77 GO terms belong to molecular functions (e.g., dynein intermediate chain binding, troponin T binding, and telomeric RNA binding). The top 30 differential enriched GO terms in each infection stage are shown in Figure S1.

**Figure 2:**
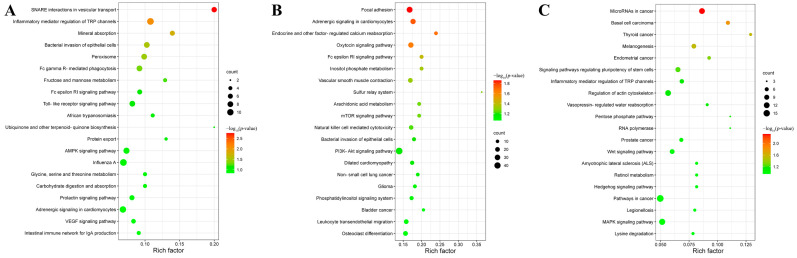
Scatter plots of the top 20 KEGG pathways of the potential targeted genes of differentially expressed miRNAs (DEmiRNAs) at (**A**) 24 h post-infection (hpi), (**B**) 96 hpi, and (**C**) 36 days post-infection (dpi) in the spleen of puppies. The rich factor is represented by the *X*-axis label, and the KEGG pathway name is displayed on the *Y*-axis label. The percentage of DEmiRNAs-targeted genes in a specific pathway is reflected in the rich factor. The dots’ colors represent the enrichment score [−log_10_(*p*-value)], with green representing low enrichment and red representing high enrichment. The number of targeted genes of DEmiRNAs in the appropriate pathway is represented by the size of the dots (bigger dots indicate larger number of DEmiRNAs targeted genes).

The potential target genes of DEmiRNAs were enriched by KEGG enrichment analysis (Table S5). The results showed that at 24 hpi, 41 potential target mRNAs (e.g., *pik3cd*, *pik3r5,* and *hmox1*) of 10 DEmiRNAs were associated with six enriched KEGG pathways (e.g., SNARE interactions in vesicular transport, inflammatory mediator regulation of TRP channels and mineral absorption). At 96 hpi, 98 potential target mRNAs (e.g., *pik3cd*, *syk,* and *nfatc1*) of 17 DEmiRNAs were associated with seven enriched KEGG pathways (e.g., focal adhesion, inositol phosphate metabolism, and vascular smooth muscle contraction). At 36 dpi, 19 potential target mRNAs (e.g., *tcf7l2*, *hpas,* and *map2k2*) of six DEmiRNAs were associated with five enriched KEGG pathways (e.g., basal cell carcinoma, melanogenesis, and microRNAs in cancer). The top 20 KEGG pathways in each infection stage are shown in Figure 2.

## 4. Discussion

The current study revealed the differential profile of splenic miRNA at three different stages after *T. canis* infection, which may have an impact on the pathology of toxocariasis. At the three different stages, overall, 496 miRNAs were identified, with 64 miRNAs being differentially expressed, including 20, 34, and 19 DEmiRNAs at 24 hpi, 96 hpi, and 36 dpi, respectively.

At 24 hpi, cfa-miR-320 was upregulated 1.43 times. A previous study showed that the miR-320 upregulation can boost the expression of inflammatory cytokines [19]. The nuclear factor of activated T cells 1 (*nfatc1*), one of the predicted target genes of cfa-miR-320, was successfully assigned to two significantly enriched GO terms in this study, such as cytoplasm and cytoplasmic part. *nfatc1* is integral for immune system development, which can induce the expression of cytokine genes in T cells [20]. The upregulation of cfa-miR-320 could play a role in resisting the invasion of *T. canis* larvae by inducing an inflammatory response or inhibiting the T-cell function by targeting *nfatc1* at the early infection stages. The downregulation of miR-193a clearly decreases the pro-inflammatory cytokines expression level [21]. At 24 hpi, cfa-miR-193a was downregulated 1.51 times. *cd40*, one of the predicted target genes of cfa-miR-193a, was successfully assigned to 33 significantly enriched GO terms, such as localization, tissue migration, and endothelial cell migration. *cd40* ligation can induce monocytes to produce several cytokines, one of which is IL-10, an anti-inflammatory cytokine [22]. The downregulation of cfa-miR-193a could attenuate the inflammatory response after *T. canis* infection by targeting *cd40*. cfa-miR-22 was downregulated 1.75 times at 24 hpi. Interferon regulatory factor 1 (*irf1*), one of the predicted target genes of cfa-miR-22, impacts myeloid lineages and lymphoid differentiation [23], the activity and maturation of NK cells, the development of macrophages, the maturation of CD8+ T cells and the maturation and differentiation of dendritic cells [24]. It is speculated that at 24 hpi, cfa-miR-22 was involved in immune activity by targeting *irf1* in the spleen of puppies after *T. canis* infection.

At 96 hpi, cfa-miR-331 is positively correlated with inflammatory response [25]. It was upregulated 1.48 times and was predicted to have five potential target genes, including *nfatc1*, myelin basic protein (*mbp*), semaphorin 7a (*sema7a*), TNF receptor superfamily member 1a (*tnfrsf1a*) and spleen associated tyrosine kinase (*syk*). Of these, at 96 hpi, GO annotation analysis showed that *mbp* was successfully assigned to two significantly enriched GO terms, such as neuron part and cellular component; *sema7a* was successfully assigned to 25 significantly enriched GO terms, such as cell growth, regulation of cell communication and positive regulation of cellular process; *tnfrsf1a* was successfully assigned to 15 significantly enriched GO terms, such as TNF-activated receptor activity, TNF binding, and oxoacid metabolic process; and *syk* was successfully assigned to 61 significantly enriched GO terms, such as kinase activity, biological adhesion, and protein kinase activity. *syk* was significantly enriched in Fc epsilon RI signaling pathway based on KEGG enrichment analyses. It was reported that *mbp* and its related transcripts are predominantly expressed in T cells [26]. A pro-inflammatory response was started by *mbp*-activated Th1 cells, increasing the expression of IFN-*γ*, TNF-*α*, -*β*, and IL-1*β*, while an anti-inflammatory response was started by *mbp*-activated Th2 cells, increasing the expression of IL-4, -10, and -13, which led to tissue damage and repair, respectively [27]. *sema7a*, present on activated T cells, reduces inflammation by stimulating cytokine production by macrophages and monocytes via a1b1 integrin [28,29]. *tnfrsf1a* is widely expressed in a variety of tissues, such as lymph nodes, spleen, thymus, lung, liver, and intestine. In parallel, in vitro, the *tnfrsf1a* gene suppresses the NF-*κ*B signaling pathway while activating the TNF signaling pathway [30,31]. Moreover, the *syk* expression is necessary for normal B-cell development and B cells activation [32]. At 96 hpi, the upregulation of cfa-miR-331 may enhance the host inflammatory response, possibly mainly by targeting *sema7a* and *tnfrsf1a*, which are negatively associated with the inflammatory response.

cfa-miR-210 was predicted to regulate two potential target genes (*syk* and *cd320*) and was upregulated 1.47 times at 96 hpi. GO annotation analysis showed that *cd320* was successfully assigned to three significantly enriched GO terms at 96 hpi, such as membrane part, membrane, and cellular component. *cd320*, FDC-signaling molecule 8D6, is a plasma membrane protein found on follicular dendritic cells (FDC) with functions as a co-stimulator to boost the stimulation of B-cell by antigen [33,34]. It was reported that *cd320* enhances IL-10-directed B-cell to plasma cell differentiation in germinal centers [35]. cfa-miR-210 negatively regulates pro-inflammatory cytokines [36], and miR-210 knockdown in hematopoietic lineage cells attenuates symptoms of parasitic diseases and limits pathogen transmission [37]. In this study, the upregulation of cfa-miR-210 indicated that the immune activity of the organism could be diminished by targeting *syk* and *cd320*, reducing the B-cell response to antigenic stimulation at 96 hpi.

At 96 hpi, cfa-miR-125a was downregulated 1.48 times. Some studies have shown that the normal growth and homeostasis of numerous immune cells are regulated by the miR-125 family [38], and miR-125a may inhibit the NF-*κ*B and Wnt/*β*-catenin pathways to lessen proliferation and inflammation [39]. TNF, one of the potential target genes of cfa-miR-125a, is negatively correlated with miR-125a [40]. The results of GO and KEGG analyses showed that TNF was successfully assigned to 103 significantly enriched GO terms at 96 hpi, such as regulation of B-cell-mediated immunity, cellular extravasation, and leukocyte tethering or rolling; and to one KEGG pathway, Fc epsilon RI signaling pathway, at 96 hpi. TNF, a multifunctional pro-inflammatory cytokine, is mainly secreted by macrophages [41] and is a key mediator and regulator of mammalian immune responses, governing the development of the immune system [42]. This information suggests that downregulated cfa-miR-125a may enable an enhanced inflammatory response in the spleen of Beagle puppies by targeting TNF.

In the present study, cfa-miR-23b, which suppressed inflammatory responses [43], was upregulated 2.21 times and 1.83 times at 96 hpi and 36 dpi, respectively. As one of the potential target genes of cfa-miR-23b, G protein-coupled receptor class c group 5 member b (*gprc5b*) was successfully assigned to 18 significantly enriched GO terms at 96 hpi, such as membrane part, enzyme regulator activity, and enzyme activator activity; and 25 GO terms at 36 dpi, such as intracellular, extracellular region and positive regulation of phosphorylation, based on GO annotation analysis. *gprc5b* regulated inflammatory response via NF-*κ*B signaling, where overexpression of *gprc5b* boosted the NF-*κ*B activation [44]. At 96 hpi and at 36 dpi, cfa-miR-23b, which may regulate *gprc5b*, was upregulated, suggesting that NF-*κ*B activation promoted by *gprc5b* may be attenuated, and inflammatory response may also be attenuated.

At 36 dpi, cfa-miR-339, which attenuated inflammation and apoptosis [45], was upregulated 1.37 times, and its regulation of immune and inflammatory activity in spleens of Beagle puppies against *T. canis* infection could be mediated by targeting the predicted target genes *mbp*, *sema7a*, *tnfrsf1a,* and *IL12b*. Among them, *IL12b* was successfully assigned to 64 significantly enriched GO terms at 36 dpi, such as defense response to protozoan, regulation of inflammatory response, and interleukin-12 alpha subunit binding. *IL12b* can serve as a growth factor for T cells and activated NK, increasing the lytic activity of NK/lymphokine-activated killer cells and inducing the production of IFN-*γ* [46]. However, upregulation of cfa-miR-339 in the present study may inhibit IL-12b function, and the phenomenon that *T. canis* infection inhibits IL-12 production was confirmed in mice experimentally infected with *T. canis* [47].

At 36 dpi, cfa-miR-206 was downregulated 1.79 times, and overexpression of miR-206 increased the inflammatory cytokines (CCL5 and IL-1*β*, -6) expression, whereas knockdown of miR-206 had the inverse effects [48]. Transcription factor 7 like 2 (*tcf7l2*), one of the potential target genes of cfa-miR-206, was successfully assigned to 55 significantly enriched GO terms at 36 dpi, such as cell-cell signaling, regulation of response to stimulus and Wnt signaling pathway; and *tcf7l2* was associated with four significantly enriched KEGG pathways, such as basal cell carcinoma, thyroid cancer, and melanogenesis, based on GO and KEGG analyses. Studies indicated that *tcf7l2* is a pivotal effector in the composition of the *tcf7l2*/*β*-catenin complex [49], which induces or represses transcription of many target genes in stress response, metabolism, and cell proliferation or death [50,51]. In the present study, downregulation of cfa-miR-206 could lead to an attenuated inflammatory response by targeting *tcf7l2*.

At 36 dpi, cfa-miR-136 was downregulated 1.91 times. The upregulation of cfa-miR-136 alleviates inflammatory injury [52]. *IL17f*, one of the potential target genes of cfa-miR-136, was successfully assigned to 15 significantly enriched GO terms at 36 dpi, such as regulation of inflammatory response, regulation of response to stimulus, and regulation of response to wounding. Additionally, *IL17f*, the T-helper 17 cells’ (Th17) hallmark effector cytokine [53], participates in both the innate and adaptive immune systems’ antimicrobial host defense and maintenance of tissue integrity [54]. Of which, *IL17f* is primarily involved in host defense by inducing neutrophilic inflammation [55] and activating NF-*κ*B and MAP kinase pathways, ultimately leading to antimicrobial peptides, matrix metalloproteinases, cytokines, and chemokines transcriptional activation, with the potential for significant immunological inflammation [56]. Downregulated cfa-miR-136 enhances immune inflammatory responses in the spleen of Beagle puppies by targeting *IL17f*.

In this study, the up- and down-regulation of DEmiRNAs ultimately may impact the immune regulation of Beagle puppies against *T. canis* infection. It can be shown that dynamic balances between pro-inflammatory and anti-inflammatory responses, and immuno-suppressive and modulatory effects induced by *T. canis* infection, and the innate and adaptive immune response of the puppies against *T. canis* infection. These findings supported earlier findings that the dynamic balance between pro-inflammatory and anti-inflammatory responses mediates the growth of *T. canis* in the definitive canine host [11,12]. However, at 24 hpi, most of the DEmiRNAs regarding immune response were downregulated, suggesting that immune activity against *T. canis* infection may be attenuated in puppies at this stage of infection, whereas at 96 hpi when *T. canis* appeared in the lungs of puppies, and at 36 dpi when *T. canis* settles in the intestinal tract after completion of tracheal migration, the host’s immune response against *T. canis* infection and mild parasite suppression essentially reached a dynamic balance, especially at 36 dpi.

## 5. Conclusions

Using RNA-seq, the present study provided a quantized and holistic analysis of spleen miRNAs of Beagle puppies after infection by *T. canis*. As the infection progresses, the research showed transcriptional changes as well as notable differences in the expression and abundance patterns of miRNAs in the spleen between infected and uninfected puppies. Our findings indicate that the expression patterns of diverse populations of miRNAs in the spleen were particular to the advancement of *T. canis* infection. Investigating these miRNAs’ functional roles should provide additional knowledge for understanding the pathogenesis of toxocariasis.

## Data Availability

The datasets supporting the results of this article have been submitted to the NCBI Sequence Read Archive (SRA) under the accession number PRJNA855014.

## Supplementary Materials

The following are available online at https://www.mdpi.com/article/10.3390/ani12192638/s1, Table S1. The primers used in the qRT-PCR experiment. Table S2. The differentially expressed miRNAs at 24 hpi, 96 hpi, and 36 dpi. Table S3. The differentially expressed miRNAs and their targeted genes. Table S4. The differential enriched Gene Ontology (GO) terms of potential target genes of the differentially expressed miRNAs. Table S5. The top 20 enriched Kyoto Encyclopedia of Genes and Genomes (KEGG) pathways of potential target genes of the differentially expressed miRNAs. Figure S1. Round enrichment chart of the top 30 enriched Gene Ontology (GO) terms of potential target genes of the differentially expressed miRNAs at (A) 24 h post-infection (hpi), (B) 96 hpi, and (C) 36 days post-infection (dpi) in Beagle dogs’ spleen infected with *Toxocara canis*. From the outside to the inside in order: the GO term ID, the number of all genes associated with the GO, the number of predicted target genes associated with the GO and the rich factor (the higher the bar, the larger the rich factor; the lower the bar, the smaller the rich factor). The color of GO term ID indicates the categories: purple indicates cellular component, yellow indicates biological process, and blue indicates molecular function; the color of the number of all genes associated with the GO indicates score [−log_10_(*p*-value)] (the shade of color represents the level of enrichment).
